# Dynamics of disease progression during treatment with Osimertinib in patients with *EGFR* T790M‐positive non‐small cell lung cancer

**DOI:** 10.1002/cam4.5926

**Published:** 2023-04-25

**Authors:** Hye Sook Kim, Kun Young Lim, Soo‐Hyun Lee, Hyae Young Kim, Youngjoo Lee, Ji‐Youn Han

**Affiliations:** ^1^ Division of Oncology/Hematology, Department of Internal Medicine Ilsan Paik Hospital, Inje University Goyang Republic of Korea; ^2^ Department of Radiology and Research Institute and Hospital National Cancer Center Goyang Republic of Korea; ^3^ Center for Lung Cancer, Research Institute and Hospital National Cancer Center Goyang Republic of Korea

**Keywords:** disease progression, *EGFR*, non‐small cell lung cancer, osimertinib

## Abstract

**Background:**

Patterns of treatment failure and subsequent treatment in non‐small cell lung cancer (NSCLC) patients treated with osimertinib are scarcely known. We analyzed the disease progression during osimertinib treatment to identify potential treatment strategies.

**Methods:**

We identified advanced NSCLC patients who commenced osimertinib treatment after progression on previous epidermal growth factor receptor (EGFR)‐tyrosine‐kinase inhibitor (TKI) from June 2014 to November 2018 from electronic records. Patients' tumor characteristics, efficacy outcomes, affected organs from radiology studies, and treatment modalities before and after osimertinib were analyzed.

**Results:**

Eighty‐four patients were included. At osimertinib initiation, bone (50.0%) and brain (41.9%) were the commonest single metastatic sites, whereas thoracic involvement (73.3%) was more frequent than bone (27.4%) or brain (20.2%) metastasis during disease progression on osimertinib. Oligo‐progressive disease (PD) and central nervous system (CNS)‐sanctuary PD were observed in 15 (17.9%) and 3 (3.6%) patients, respectively. Most patients without brain metastasis (BM) at osimertinib initiation remained BM‐free (46/49, 93.9%), and 60% of patients (21/35) with pre‐existing BM showed intracranial disease control despite extracranial PD. The resistance mechanisms to osimertinib were explored in 23 patients (27.4%), and T790M‐loss was observed in 14 patients (60.9%) who had worse survival outcomes than those without T790M‐loss (progression‐free survival, 5.4 vs. 16.5 months, *p* = 0.02; overall survival, not reached, *p* = 0.03).

**Conclusion:**

PD during osimertinib treatment occurred preferentially in the thorax and pre‐existing sites. Extracranial PD prevailed over intracranial PD regardless of baseline BM and prior brain radiation. These results support osimertinib's intracranial efficacy and may guide treatment strategies for *EGFR‐*mutated NSCLC with BM.

## 1 INTRODUCTION

Non‐small cell lung cancer (NSCLC) is a leading cause of cancer‐related death. However, recent developments in molecular genetics have revealed multiple actionable oncogenic drivers in NSCLC, and treatment outcomes in patients with NSCLC with actionable mutations have improved in the last two decades.^1^ Currently, epidermal growth factor receptor (EGFR)—tyrosine kinase inhibitors (TKIs) are the standard treatment for patients with locally advanced or metastatic NSCLC with EGFR mutations. When failure of first‐ or second‐generation EGFR‐TKIs occurs after approximately 1 year of progression‐free survival (PFS), an EGFR T790M mutation has been proved to contribute up to 50% of resistance mechanism. Osimertinib is a third‐generation EGFR‐TKI with clinically proven efficacy against sensitizing and T790M‐resistant *EGFR* mutations.^2^

Sites of progressive disease (PD) and symptoms among patients with acquired resistance to the first‐ or second‐generation EGFR‐TKIs vary.^3^, ^4^ The primary lung lesion is the most frequent PD site, and central nervous system (CNS) failure incidence is reported at up to 40% in patients treated with EGFR‐TKI.^4^, ^5^ Osimertinib has demonstrated remarkable activity against CNS metastases compared to that of control arms in both first‐ and second‐ or third‐line therapy for EGFR‐mutant NSCLC.^6^, ^7^ However, the development of resistance to osimertinib is inevitable in most patients. Furthermore, little is known about the pattern of osimertinib treatment failure.

Clinically authentic groups of EGFR‐TKI failure have been suggested, including dramatic, gradual, and local progression, according to the rapidity of PD.^8^ Tumors with slow or local progression may have more indolent biology, making metastasis‐directed local ablative therapy (LAT) a viable treatment option. As a result, the concept of oligometastasis or oligoprogression was recently adopted for patients with disease progression in a limited number of organs.^9^, ^10^, ^11^ Contrary to the classic theory of a change to systemic therapy with all types of treatment failure, a treatment strategy incorporating aggressive LAT while maintaining EGFR‐TKI therapy has been attempted to eradicate the locally‐remaining or PD in metastatic EGFR‐mutated NSCLC.^12^, ^13^ Regarding tumors that have developed resistance to osimertinib, the aforementioned innovative treatment strategies are essential.

Despite osimertinib being the treatment of choice for EGFR T790M‐positive NSCLC, the pattern of osimertinib resistance and the appropriate subsequent treatment guidelines are limited in the real‐world setting.^14^, ^15^, ^16^, ^17^, ^18^ This study aimed to determine the effective treatment direction after osimertinib administration by analyzing the pattern of PD in patients with NSCLC who were treated with osimertinib after first‐line EGFR‐TKI therapy.

## 2 MATERIALS AND METHODS

This retrospective study included patients with NSCLC who commenced osimertinib treatment between June 2014 and November 2018 at the National Cancer Center in Korea. Clinical and demographic data, including age, sex, smoking status, performance status, and treatment modality, were collected from the medical record system. The disease extent was assessed using radiological reports. The study protocol and waiver of informed consent were approved by the institutional review board of the National Cancer Center (No. NCC 2019‐0305).

Patient inclusion criteria were as follows: (1) recurrent or initially advanced or metastatic NSCLC with documented EGFR‐TKI sensitizing mutations, (2) treatment with osimertinib after disease progression following first‐ or second‐generation EGFR‐TKI treatment, (3) disease progression during osimertinib treatment, and (4) performance of imaging studies of affected organs and brain upon disease progression. Patient exclusion criteria were as follows: (1) treatment with other third‐generation EGFR‐TKIs prior to osimertinib treatment, (2) premature osimertinib treatment for less than 4 weeks, and (3) no T790M mutation confirmed after the failure of the previous EGFR‐TKI. Disease response during osimertinib treatment was evaluated using RECIST 1.1. Clinical variables, including anticancer treatment, results of imaging studies, and mutation profiles, were collected. Imaging modalities included computed tomography (CT), magnetic resonance imaging (MRI), whole‐body bone scans, and fluorodeoxygenase‐positron emission tomography/computed tomography (FDG‐PET/CT). After disease progression during osimertinib treatment was confirmed, the number of organs with metastases and the lesions in the organs from the imaging studies, additional EGFR mutation analysis, and types of subsequent treatment were detailed whenever possible. All patients were followed up until the date of death or March 2020, whichever occurred first.

Patterns of PD during osimertinib treatment were classified as follows: (1) CNS‐ sanctuary PD, (2) oligoprogression (oligo‐PD), and (3) systemic PD. CNS‐sanctuary PD represents isolated CNS failure in the absence of systemic PD or leptomeningeal seeding. Oligo‐PD was defined as disease progression of no more than five lesions in no more than three organs when the remaining metastases were well‐controlled with systemic therapy. PD with pleural or pericardial effusion and ascites was excluded from oligo‐PD. The involvement of regional lymph nodes, including the hilar, mediastinal, and supraclavicular lymph nodes, was considered thoracic disease. PD other than CNS‐sanctuary PD and oligo‐PD were considered systemic PD.

Chi‐square test, Fisher's exact test, and Mann–Whitney *U* test were used, when appropriate, to compare clinical and biological features among different groups. PFS and overall survival (OS) were determined using the Kaplan–Meier method, and survival curves were compared using the log‐rank test. Odds ratios (ORs) and 95% confidence intervals (CIs) were estimated using a logistic regression model, and hazard ratios (HRs) and 95% CIs were estimated using the Cox proportional‐hazards model. All statistical tests were two‐sided, and *p*‐values ≤0.05 were deemed statistically significant. Statistical analyses were performed using IBM SPSS Statistics for Windows, version 25 (IBM Corp.).

## 3 RESULTS

### 3.1 Patient characteristics

Of the 132 patients screened for eligibility, 84 were included in the final analysis. In total, 48 patients were excluded because they were previously treated with another 3rd generation EGFR‐TKI other than osimertinib (*n* = 27), or had no documented T790M mutation after previous EGFR‐TKI (*n* = 10), or had no adequate brain imaging at the beginnings or end of the osimertinib treatment (*n* = 6), or treated with osimertinib less than 4 weeks (*n* = 4), or had no EGFR‐TKI before osimertinib (*n* = 1). As shown in Table 1, the median age at the start of osimertinib treatment was 62.4 (range, 40.0–85.0) years. Most of the patients were women (59.5%) and never‐smokers (59.5%). The patients had Eastern Cooperative Oncology Group (ECOG) performance scores ranging from 0 to 2. After excluding six scores due to metastatic cancer, patients had a median Charlson Comorbidity Index of 1 (range, 0–7). Most tumors (97.6%) exhibited adenocarcinoma pathology, while adenosquamous and squamous cell carcinoma occurred in one case each. Patients had been treated with first‐ or second‐generation EGFR‐TKIs, including gefitinib (*n* = 33, 39.3%), erlotinib (*n* = 43, 51.2%), or afatinib (*n* = 6, 7.1%). The remaining two patients were treated with erlotinib and bevacizumab according to the clinical trial protocol. After disease progression during initial EGFR‐TKI treatment, 58 (69%) patients were subsequently treated with osimertinib. Thirty patients (35.7%) were treated with cytotoxic chemotherapy before osimertinib, and the median number of chemotherapy regimens was 1.5. Sixteen patients (19.1%) received three or more chemotherapy regimens before osimertinib.

**TABLE 1:** Patient characteristics.

Characteristics	Value (*N* = 84)	%
Age (median ± SD, range, years)	62.4 ± 9.8, 40.0–85	
Gender		
Female/Male	50/34	59.5/40.5
Performance status (ECOG)		
0	22	31.0
1	31	43.7
2	20	25.4
Smoking history		
Never	50	59.5
Former	32	38.1
Current	2	2.4
Histology		
Adeno	82	97.6
Adenosquamous	1	1.2
Squamous	1	1.2
Baseline EGFR mutation		
Del 19	45	53.6
L858R	25	29.8
G719X	3	3.6
T790M with other sensitizing mutation	3	3.6
Others	8	9.5
Stage		
Recurred	11	13.1
Initially metastatic	73	86.9
Initial EGFR‐TKI		
Gefitinib	33	39.3
Erlotinib	43	51.2
Afatinib	6	7.1
Others	2	2.4
Tx. After PD during initial EGFR‐TKI		
Osimertinib	58	69.0
Cytotoxic	24	28.6
No. of previous systemic treatments before osimertinib		
1	53	63.1
2	15	17.9
≥3	16	19.1
Previous cytotoxic chemotherapy before osimertinib		
Yes	30	35.7
No	54	64.3

Abbreviations: ECOG, Eastern Cooperative Oncology Group; EGFR, epidermal growth factor receptor; PD, progressive disease; SD, standard deviation; TKI, tyrosine kinase inhibitor.

### 3.2 Distribution of disease and pattern of disease progression

**FIGURE 1:**
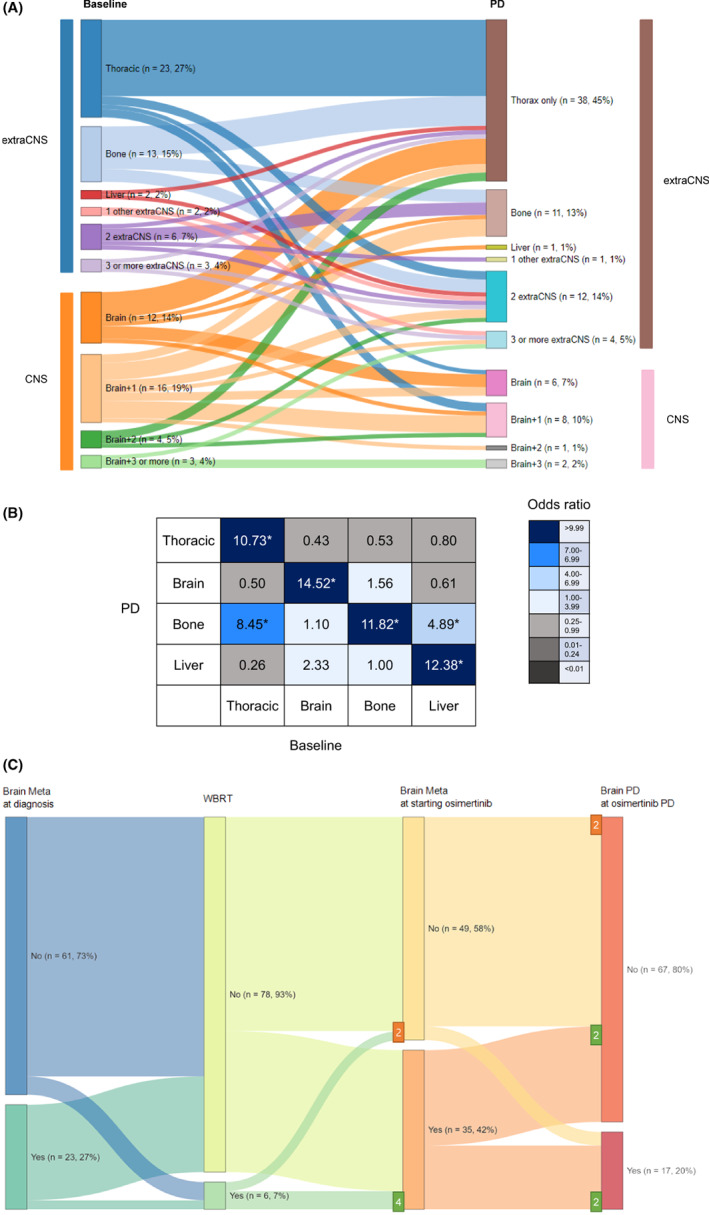
Progressive disease (PD) pattern during osimertinib treatment (A) Sankey diagram showing PD organs as assigned in the PD from osimertinib and per involved organ at the initiation of osimertinib treatment. The right column shows that the first node was split based on involved organs at the start of osimertinib treatment. The second node was split by the PD organs. Involved organs are categorized according to the central nervous system (CNS) metastasis (baseline blue bar encompass over extra‐CNS metastasis and orange bar over CNS metastasis vs. PD brown bar over extra‐CNS metastasis and pale pink bar over CNS metastasis). Diagram created using Sankey diagram web tool (available at: https://www.displayr.com/). (B) Odds ratio comparison among different metastatic combinations. The asterisk indicate statistically significance of the odds ratio. (C) Sankey diagram representing flows of patients from diagnosis to endpoint. Flows are presented according to the brain metastasis at initial diagnosis of advanced non‐small cell lung cancer (NSCLC), whole brain radiation therapy (WBRT), brain metastasis at starting osimertinib treatment, and brain PD at PD from osimertinib treatment. Numbers represent the flow of patients who were treated with WBRT prior to osimertinib treatment.

At the start of osimertinib, the incidence of bone metastasis (42%, 50.0%) was highest among all distant organs with metastases. The second most common organ with metastases was the brain (35%, 41.7%). Many patients had metastasis to more than one organ, and patients with metastases to more than two organs were stratified by brain metastasis (BM) (Figure 1A left column, Table S1). Twenty‐three (27.4%) patients had disease confined to the thorax, which indicates NSCLC metastasis to the contralateral lung, pleura, or intrathoracic and supraclavicular lymph nodes. The two leading single site organs of metastasis were the bones (*n* = 13, 15.5%) and brain (*n* = 12, 14.3%). Patients with isolated liver metastases (*n* = 2, 2.4%) and metastases at other sites were rare (one patient each with peritoneal and adrenal gland metastases). For the combination of metastases to the brain, the number of patients with different organ sites of metastasis was classified as one (*n* = 16, 19.0%), two (*n* = 4, 4.8%), and three or more (*n* = 3, 3.6%). For combination metastases involving organs other than that of the brain, the proportion of patients with two and three or more organ sites of metastasis was 7.1% and 3.6%, respectively.

Changes in metastatic lesions during osimertinib treatment are plotted as an alluvial diagram (Figure 1A). While the fraction of PD in multiple organs other than the brain increased from the baseline extra‐CNS metastasis (*n* = 67, 79.8%; brown bar from *n* = 49, 58.3%; blue bar), the fraction of PD involving BM decreased (*n* = 17, 20.2%; pale pink bar from *n* = 35, 41.7%; orange bar) from the baseline BM. The most frequent PD site was the thorax (63, 75.0%), which means PD occurred in the thorax, with or without PD in other organs. PD confined to only the thoracic region was observed in 38 (45.2%) patients. The other single organs that were frequently involved in PD were the bones (*n* = 23, 27.4%) and brain (*n* = 17, 20.2%). To better understand the interactions among the organs that were sites of metastasis from baseline to PD, the ORs of each combination were compared (Figure 1B). Each metastatic lesion at PD (vertical column) was preferentially related to the metastatic lesion at baseline (horizontal column); thoracic‐only disease was related to PD in the thorax (OR 10.73, 1.35–85.40, *p* < 0.01); BM was related to PD in the brain (OR: 10.22, 2.65–39.41, *p* < 0.01); bone metastasis was related to PD in the bones (OR: 11.82, 3.15–44.30, *p* < 0.01); and liver metastasis was related to PD in the liver (OR:12.38, 2.87–53.42, *p* < 0.01) and bones (OR 4.89, 1.47–16.30, *p* = 0.02).

Regarding the patterns of PD during osimertinib treatment, CNS sanctuary PD, oligo PD, and systemic PD were observed in 3 (3.6%), 15 (17.9%), and 67 patients (79.8%), respectively. When comparing clinicopathologic factors between oligo PD and systemic PD, the proportion of patients who received subsequent osimertinib treatment immediately after the previous EGFR‐TKIs seemed higher in oligo PD than in systemic PD (86.7% vs. 65.2%, *p* = 0.08, Table 2). More exposure to cytotoxic chemotherapy before osimertinib showed a trend toward systemic PD (20.0% vs. 37.9%, *p* = 0.05, Table 2).

**TABLE 2:** Patient characteristics stratified by PD type.

Characteristics	Systemic PD (*N* = 66)	Oligo PD (*N* = 15)	*p*
Age (median ± SD, range, years)	62.5 ± 10.0, 40.0–85.0	62.4 ± 8.3, 53.0–83.0	0.42
Gender, female/male	42 (63.6)/24(36.4)	6(40.0)/9(60.0)	0.09
Smoking history			0.76
Never	41 (62.1)	9 (60.0)	
Former	23 (34.8)	6 (40.0)	
Current	2 (3.0)	0	
Smoking pack‐year (mean)	7.5	10.4	0.56
Baseline EGFR mutation			
Del 19	34 (51.5)	10 (66.7%)	0.50
L858R	22 (33.3)	2 (13.3%)	
G719X	2 (3.0)	1 (6.7%)	
T790M with other sensitizing mutation	3 (4.5)	0	
Stage			
Recurred	7 (10.6)	3 (20.0)	0.32
Initially metastatic	59 (89.4)	12 (80.0)	
Initial brain metastasis			
Yes	18 (27.3)	4 (26.7)	0.96
No	48 (72.7)	11 (73.3)	
Tx. After PD during initial EGFR‐TKI			
Osimertinib	43 (65.2)	13 (86.7)	0.08
Cytotoxic	22 (33.3)	1 (6.7)	
No. of previous systemic treatments before osimertinib			
1	40 (60.6)	12(80.0)	0.05
2	13 (19.7)	1 (6.7)	
≥3	13 (19.7)	2 (13.3)	
Previous cytotoxic chemotherapy before osimertinib			
Yes	25 (37.9)	3 (20.0)	0.19
No	41 (62.1)	12 (80.0)	
Involved organ at starting Osimertinib			0.50
Thoracic only	16 (24.2)	7 (46.7)	
Bone meta only	10 (15.2)	3 (20.0)	
Brain meta only	7 (10.6)	3(20.0)	
Liver meta only	2 (3.0)	0	
1 Other extra CNS meta only	2 (3.0)	0	
2 extraCNS meta	6 (9.1)	0	
3 or more extraCNS meta	3 (4.5)	0	
Brain meta+1	14 (21.2)	3 (20.0)	
Brain meta+2	3 (4.5)	1 (6.7)	
Brian meta+3 or more	3 (4.5)	0	
PD organ			0.21
Thorax	26 (39.4)	12 (80.0)	
Bone	8 (12.1)	3 (20.0)	
Brain	3 (4.5)	0	
Liver	1 (1.5)	0	
1 other extraCNS	1 (1.5)	0	
2 extraCNS	12 (18.2)	0	
3 or more extraCNS	4 (6.1)	0	
Brain+1	8 (12.1)	0	
Brain+2	1 (1.5)	0	
Brain+3	2 (3.0)	0	
Osimertinib resistant mutation			0.24
T790M loss	9 (47.4)	1 (33.3)	
T790M loss/additional mutation	1 (5.3)	0	
No T790 loss	6 (31.6)	0	
No T790 loss/ additional mutation	3 (15.8)	2 (66.7)	

Abbreviations: CNS, central nervous system; ECOG, Eastern Cooperative Oncology Group; EGFR, epidermal growth factor receptor; PD, progressive disease; SD, standard deviation; TKI, tyrosine kinase inhibitor.

The pattern of PD concerning BM is shown in Figure 1C. Twenty‐three (27%) patients had metastasis to the brain at the time of NSCLC diagnosis. Before starting the osimertinib treatment, more BM appeared, and six (7%) patients were treated with whole‐brain radiation therapy (WBRT). When starting osimertinib treatment, 35 patients (42%) had BM. Among the six patients who were treated with WBRT, two had no evidence of intracranial metastasis in the MRI brain at commencing osimertinib. During osimertinib treatment, 14 patients (40%) with BM experienced progression of BM, of whom 10 had extracranial disease (71.4%), and 4 had no extracranial disease (23.5%). Among patients who showed PD in the brain during osimertinib treatment, 17.6% (3/17) patients had no previous BM. Among the six patients who underwent WBRT before osimertinib treatment, two with no evidence of BM at the start of osimertinib therapy showed no intracranial progression. Among the other four patients treated with WBRT before osimertinib, two had brain PD, and the other two had multiple PD in extracranial organs. Due to the small number of patients treated with WBRT, statistical computation of the PD risk reduction from WBRT was not feasible. However, the impact of WBRT on the development of CNS PD during therapy was evaluated with intracranial time to progression (TTP, Figure S1), and intracranial TTP in patients treated with WBRT (*n* = 6, median 13.0 months) was not significantly longer than that in patients not treated with WBRT (*n* = 78, median not reached, *p* = 0.44).

### 3.3 Treatment outcomes of osimertinib and subsequent treatments after PD

**FIGURE 2:**
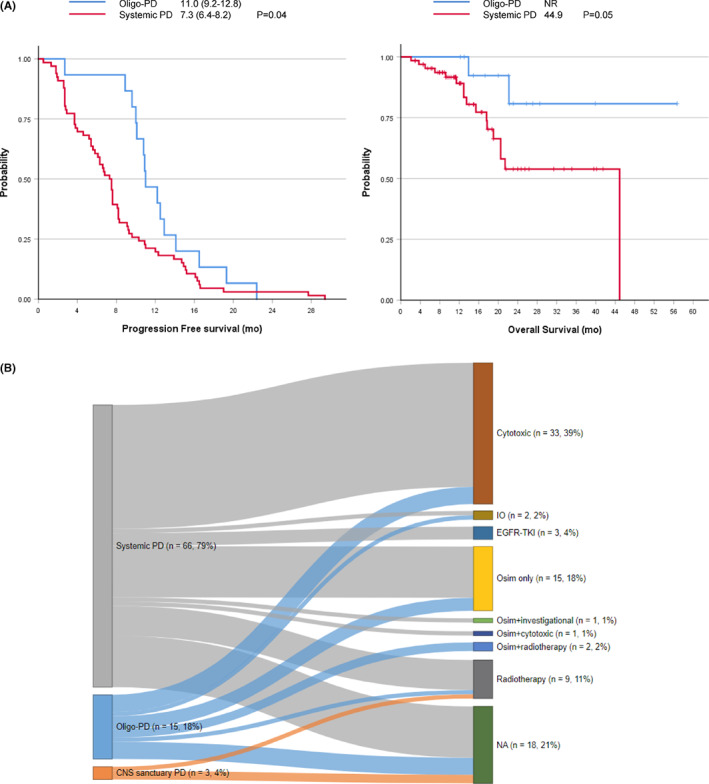
Clinical outcomes according to the types of progressive disease (PD) (A) Kaplan–Meier curves comparing the progression‐free survival (PFS) (left) and overall survival (OS) (right) of patients with oligo‐PD and systemic PD (red). Patients with oligo‐PD (blue) had significantly longer PFS with osimertinib than patients with systemic PD (red). Tick marks denote censored data. (B) Sankey diagram representing the flow of treatment after PD from osimertinib treatment per type of PD.

Osimertinib yielded an objective response rate of 69.0% (58/84) and a disease control rate of 86.9% (73/84) (Table 3). The median PFS was 8.1 months (95% CI: 6.8–9.4), and the OS was 44.9 months (95% CI: 13.0–76.9). To determine the impact of the type of PD, we compared the treatment outcomes between oligo‐PD and systemic PD (Figure 2A). The median PFS was longer in the oligo‐PD subgroup (11.0 months, 95% CI 9.2–12.8) than in the systemic PD subgroup (7.3 months, 95% CI 6.8–9.4 log‐rank *p* = 0.04). Among the 84 patients included, 19 (22.6%) had died by the time of this analysis, including 2 and 17 patients in the oligo‐PD and systemic PD subgroups, respectively. The median OS was not reached in the oligo‐PD subgroup, and it was 44.9 months in the systemic PD subgroup (*p* = 0.05).

**TABLE 3:** Treatment outcomes of osimertinib.

	Number of cases	%
Response to treatment (*N* = 84)		
Partial response	58	69.0
Stable disease	15	17.9
Disease progression	11	13.1
Objective response	58	69.0
Disease control	73	86.9
Post‐osimertinib treatment (*N* = 66)		
Osimertinib maintenance	19	28.8
Osimertinib only	15	22.7
Osimertinib with radiation	2	3.0
Osimertinib with other agents	2	3.0
Systemic treatment other than osimertinib	38	57.5
Cytotoxic chemotherapy	33	50.0
EGFR TKI	3	4.5
Checkpoint inhibitor	2	3.0
Radiation	9	13.6

Abbreviations: EGFR, epidermal growth factor receptor; TKI, tyrosine kinase inhibitor.

After developing PD during osimertinib treatment, 66 of 84 patients (78.6%) were subsequently treated with other modalities (Table 3). Most patients (57/66, 86.4%) were managed with systemic treatment, and osimertinib was continued in 19 patients (28.8%), while another EGFR‐TKI, cytotoxic chemotherapy, and checkpoint inhibitors were administered in 3 (4.5%), 33 (50.0%), and 2 (3.0%) patients, respectively. Nine (13.6%) patients were treated with radiation therapy, and two (3.0%) patients were treated with radiation concurrently with osimertinib maintenance. The median treatment duration of post‐progression osimertinib treatment was 5.3 months (range 1.2–35.9 months). Among those who continued osimertinib after PD, two patients were also treated with other systemic agents (one patient with cytotoxic chemotherapy and the other with the investigational agent everolimus).

The treatment pattern stratified by the predefined subtype is plotted in Figure 2B. Patients with systemic PD (12/66, 18.2%), oligo‐PD (4/15, 26.7%), or CNS sanctuary PD (2/3, 66.7%) did not receive further treatment beyond osimertinib (green bar). The proportion of local and systemic treatments was significantly different between the types of PD (*p* = 0.04). Patients with systemic PD (47/54, 87.0%) received systemic treatment most frequently. They were administered post‐progression osimertinib with (2/54, 3.8%) or without (12/54, 22.2%) other agents, or switched to other EGFR‐TKIs (3/54, 5.6%), cytotoxic (29/54, 53.7%), and checkpoint inhibitors (1/54, 1.9%). Some patients with systemic PD (7/54, 13.0%) underwent radiation therapy (two patients were treated with WBRT and five with palliative radiation to the bone). The subsequent treatment courses for patients with oligo‐PD comprised systemic therapy (8/11, 53.3%), post‐progression osimertinib (3/11, 27.3%), cytotoxic chemotherapy (4/11, 36.4%), and checkpoint inhibitors (1/11, 9.1%). Radiation therapy was administered with (2/11, 18.2%) or without (1/11, 9.1%) osimertinib. Moreover, radiation therapy was administered to the primary lung tumors in two patients with osimertinib and to the bone in one patient.

### 3.4 Mechanisms of resistance to osimertinib

At diagnosis, most tumors (77/84, 90.5%) showed sensitizing *EGFR* mutations including exon 19 deletion (45/73, 61.6%), L858R mutation (25/73, 34.2%), G719X mutation (3/73, 4.1%), and T790M with other sensitizing mutations (3/73, 4.1%). T790M mutation was confirmed in all patients before osimertinib treatment was started, using either tissue (74/84, 88.1%) or plasma (10/84, 11.9%) samples; concomitant biomarkers were found in nine patients (10.7%) with PD‐L1 overexpression.

**FIGURE 3:**
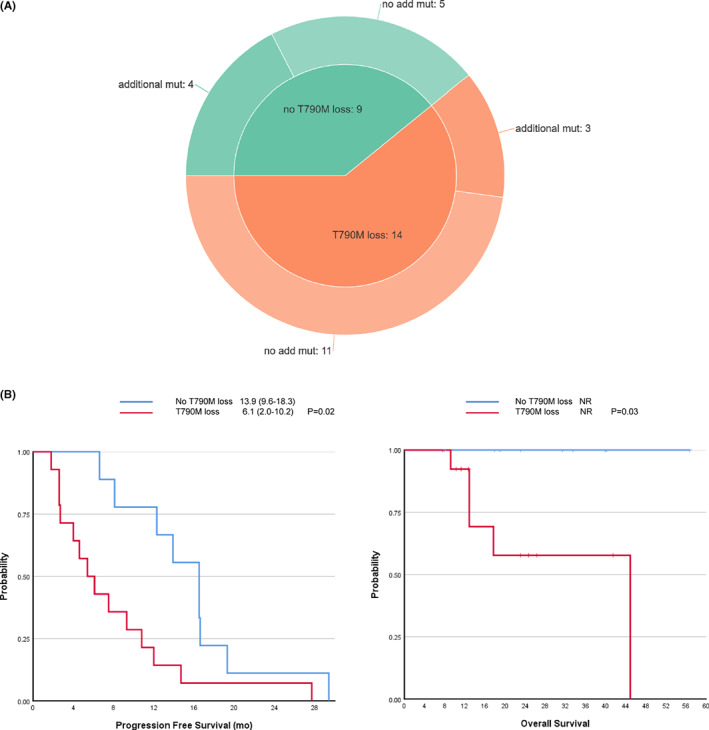
Resistance mechanisms detected after the failure of osimertinib and survival outcomes of patients according to the loss of T790M. (A) Distribution of acquired resistance mechanism (B) Kaplan–Meier curves comparing the progression‐free survival (PFS) and overall survival (OS) of patients with and without T790M loss. Tick marks denote censored data.

After developing PD on osimertinib treatment, 23(27.4%) patients underwent an additional *EGFR* mutation analysis using tissue (20/23, 87.0%) or blood (3/23, 13.0%) samples. Genetic analysis was performed with either direct sequencing (15/23, 65.2%) or next‐generation sequencing (8/23, 34.8%). Pre‐existing *EGFR* sensitizing mutations persisted after the onset of osimertinib resistance in most cases (21/23, 91.3%). Some tested samples showed a T790M loss (14/23, 60.9%), whereas others did not (9/23, 39.1%, Figure 3A). Among the patients with a T790M loss, 11 (76.9%) had no additional genetic aberrations. Three (23.1%) specimens with a T790M loss had additional mutations, including one with an *EGFR* and *AKT1* amplification and a *PDGFR* mutation (p.D1033V), one had a *CTNNB1* (p.D32Y) and a *BRAF* (p.V600E) mutation, and the other exhibited transformation to squamous cell carcinoma and contained a *TP53* mutation (p.V73fs). In the nine samples with no T790M loss after osimertinib resistance, five had no additional mutations, and the other four had other mutations. There was a histological transformation from adenocarcinoma to large‐cell neuroendocrine carcinoma among the five samples without a T790M loss or additional mutations. Other genetic aberrations included *TP53* mutations (p.L194F, p.R273C, p.Y220C, 3/5), an *ATM* mutation (p.K92T, 1/5), a MET mutation (1/5, p.T646K), (1/5), and EGFR, EZH2, ERBB2, ERBB3, FGFR3, and GNA11 amplification (1/5) in the four samples without a T790M loss.

The T790M positivity at the time of progression to osimertinib was analyzed based on clinical factors (Table 4). Previous treatments before osimertinib including cytotoxic chemotherapy, types of EGFR‐TKI, and subsequent treatment after failure of previous EGFR‐TKI and BM at initial diagnosis and at the start of osimertinib did not differ according to the T790M status at osimertinib resistance. The types of PD and involved organs at PD were not different depending on the T790M status. Interestingly, patients without a T790M loss showed better response to osimertinib than patients with a T790M loss (PR/SD 9/0 vs. 8/6, *p* = 0.05), and patients without a T790M loss showed significantly longer PFS to osimertinib (median 16.5 vs. 5.4, *p* = 0.02, Figure 3B) and OS (median not reached, *p* = 0.03, Figure 3B) than patients with a T790M loss.

**TABLE 4:** Comparison of clinical factors stratified by T790M status at osimertinib PD (*N* = 23).

Characteristics	No T790M loss (*N* = 9)	T790M loss (*N* = 14)	*p*
Age (mean ± SD, range, years)	68.2 ± 2.9	56.6 ± 2.8	0.01
Gender, female/male	7/2	9/5	0.66
Smoking history			
Never	7 (77.8)	9 (64.3)	0.66
Former	2 (22.2)	5 (35.7)	
Smoking Pack‐year (mean)	0.6 ± 0.6	4.3 ± 2.0	0.10
Baseline EGFR mutation			
Del 19	6 (66.7)	7 (50.0)	0.25
L858R	3 (33.3)	7 (53.8)	
Initial brain metastasis			
Yes	0 (0.0)	5 (35.7)	0.12
No	9 (100.0)	9 (64.3)	
Initial EGFR TKI			
Gefitinib	1 (11.1)	4 (28.6)	0.40
Erlotinib	8 (88.9)	9 (64.3)	
Afatinib	0 (0.0)	1 (7.1)	
Subsequent treatment after initial EGFR‐TKI			
Osimertinib	5 (55.6)	11 (78.6)	0.32
Other EGFR‐TKI	1 (11.1)	0 (0.0)	
Cytotoxic	3 (33.3)	3 (21.4)	
Previous cytotoxic chemotherapy before osimertinib			
Yes	4 (44.4)	4 (28.6)	0.66
No	5 (55.6)	10 (71.4)	
Involved organ at starting osimertinib			
Thoracic only	4 (44.4)	2 (14.3)	0.16
Bone metastasis	4 (44.4)	8 (57.1)	0.68
Brain metastasis	1 (11.1)	7 (50.0)	0.09
Best response			
PR	9 (100.0)	8 (57.1)	0.05
Stable Disease	0 (0.0)	6 (42.9)	
PD organ			
Thorax only	6 (66.7)	7 (50.0)	0.25
Bone	3 (33.3)	6 (42.9)	0.99
Brain	0 (0.0)	2 (14.3)	0.50
PD type			
OligoPD	2 (22.2)	1 (7.1)	0.44
CNS sanctuary PD	0 (0.0)	1 (7.1)	
Systemic PD	7 (77.8)	12 (85.7)	

Abbreviations: CNS, central nervous system; EGFR, epidermal growth factor receptor; PD, progressive disease; SD, standard deviation; TKI, tyrosine kinase inhibitor.

## 4 DISCUSSION

In this study, we evaluated the pattern of PD during and after osimertinib treatment in real‐world practice. We found that PD preferentially occurred in the previously affected extracranial organs. A small but significant number of patients showed oligo‐PD, which could benefit from LAT. Post‐osimertinib treatment included systemic therapy in most cases and LAT in selected cases. This study provides insights into the current practice patterns for patients who need further treatment beyond osimertinib in a real‐world setting.

This study demonstrated that PD during osimertinib treatment was confined to the originally involved organs. In line with previous trials that reported new lesions in 26% and 31% (AURA3 and AURA2 studies, retrospectively) of PD cases during osimertinib treatment, a recent retrospective study reported that most patients with an *EGFR* mutant NSCLC treated with osimertinib experienced their first progression in the initial sites of the disease, particularly in the thorax.^2^, ^14^, ^19^, ^20^, ^21^ These results suggest that PD during osimertinib treatment in *EGFR* mutant tumors most likely occurs in organs with a heavy tumor burden at the beginning of treatment. Contrary to the high rate of PD in extracranial organs, newly developed intracranial metastases were infrequent in this study. The frequency of intracranial failure of patients treated with osimertinib is known to be lower than that of those treated with older generation EGFRT‐TKIs because of the enhanced intracranial activity of osimertinib.^22^ In this study, the rate of patients with newly developed CNS metastases (8.6%) was low, just like that of the AURA2 study (8%). Moreover, WBRT before osimertinib administration was not associated with survival benefits in terms of intracranial TTP. As osimertinib is an active therapy for BM, WBRT can be reserved for symptomatic patients only.

Due to their superior potency over traditional cytotoxic chemotherapy, TKIs overcome some novel challenges in the therapeutic paradigm of *EGFR*‐mutant NSCLC, such as slow progression or oligo PD.^23^ Previously, the frequency of oligo‐PD was reported to be around 20% during treatment with older generation EGFR‐TKIs and up to 70% during treatment with osimertinib.^18^, ^19^, ^24^ The proportion of oligo‐PD (17.9%) and that of the combination of oligo‐PD with CNS sanctuary PD (21.4%) was relatively low in this study. In our study, although the definition of oligo‐PD is similar in terms of the number of PD lesions (no more than five) to that of other studies, we chose to limit the number of involved organs to no more than three for a better selection of candidates for the local treatment. Most patients with oligo‐PD showed involvement in only one organ (in the thorax) and either the lung or lymph node, except for one patient who showed PD with lung and brain lesions. A study that defined PD as no more than three lesions in a limited area reported a similar proportion of local progression (23.1%)^21^ Definition of oligo‐PD usually relies on radiologic patterns of disease progression with a limited number of metastatic sites; however, the associated symptoms and rapidity of PD should be considered when classifying PD. Further studies should consider how to classify oligo‐PD to guide therapeutic approaches.

Currently, concerning post‐osimertinib treatment, the focus is being placed on targeted therapies according to resistance mechanisms.^25^, ^26^ However, when there is no readily available target, innovative strategies are needed with progression during osimertinib, such as post‐progression maintenance osimertinib and LAT.^27^, ^28^ From Swiss retrospective studies,^18^ the proportions of patients who were treated with osimertinib maintenance and post‐osimertinib radiation after PD were 30.8% (8/26) and 23.1% (6/26), respectively. The rate of post‐PD osimertinib use in this study (28.8%) is similar, and more patients with oligo‐PD were treated with post‐PD osimertinib. Local treatment with a curative aim was limited to three cases from the oligo‐PD subgroup, and radiation was administered for lung and bone lesions in two and one patient, respectively. Although survival outcomes differed according to the type of PD, determining the survival benefit of different treatments, including LAT, is beyond the scope of this study. While previous retrospective studies have shown a marginal benefit of LAT in combination with post‐PD osimertinib and cytotoxic chemotherapy,^18^, ^21^, ^27^ the clinical impact of post‐PD treatment awaits further verification.

Tumors that acquire resistance to osimertinib exhibit various survival mechanisms, including the C797S mutation, T790M loss, and other novel ones. Among recent investigations on the impact of resistance mechanisms, several studies have shown that the loss of T790M results in shorter PFS during osimertinib treatment.^15^, ^16^, ^29^, ^30^ In this study, the treatment outcomes were similarly poorer in patients with a T790M loss than in those with other resistance mechanisms. However, treatment outcomes following osimertinib resistance could not be compared due to the small number of patients. Further investigations of treatment strategies to overcome osimertinib resistance based on the various molecular resistance mechanisms are needed.

Our study is a retrospective analysis; thus, it presents limitations. However, we found that disease progression during osimertinib treatment preferentially involved previously affected organs. The thoracic region showed the highest prevalence of PD, while brain PD was relatively uncommon. Finally, systemic PD was more common than oligo‐PD or CNS‐sanctuary PD, which was associated with the interval between osimertinib treatment and prior EGFR‐TKI therapy.

## 5 CONCLUSION

Our study showed that disease progression during osimertinib treatment preferentially involved the previously affected organs. The thoracic region most frequently showed PD, and brain PD was relatively uncommon, while systemic PD was more common than oligo‐PD or CNS sanctuary PD. We have also presented the various treatment options. When targeted therapy based on resistance mechanisms is unavailable, treatment strategies such as cytotoxic chemotherapy, osimertinib maintenance, and radiation should be considered.

## AUTHOR CONTRIBUTIONS

**Hye Sook Kim:** Data curation (equal); formal analysis (equal); investigation (equal); methodology (equal); project administration (equal); software (equal); supervision (supporting); validation (equal); visualization (equal); writing – original draft (equal); writing – review and editing (equal). **Kun Young Lim:** Data curation (equal); investigation (equal); resources (equal); validation (equal). **Soo‐Hyun Lee:** Data curation (supporting); investigation (supporting); resources (equal); validation (equal). **Hyae Young Kim:** Data curation (equal); investigation (equal); resources (equal); validation (equal). **Youngjoo Lee:** Conceptualization (equal); data curation (supporting); investigation (equal); project administration (supporting); resources (equal). **Ji‐Youn Han:** Conceptualization (lead); data curation (equal); formal analysis (equal); funding acquisition (lead); investigation (equal); methodology (equal); project administration (lead); resources (equal); supervision (lead); validation (equal); writing – review and editing (equal).

## FUNDING INFORMATION

This study was partly supported by a National Cancer Center Research Grant 2210551‐1. The study sponsor had no role in the study design, collection, analysis, and interpretation of data, writing of the report, and in the decision to submit the paper for publication.

## CONFLICT OF INTEREST STATEMENT

The authors declare that they have no conflicts of interest.

## ETHICS STATEMENT

The retrospective study protocol and waiver of informed consent was approved by the appropriate ethics review board (approval no. NCC 2019‐0305).

## Supporting information


Table S1.

Figure S1.

## Data Availability

The data underlying this article will be shared on reasonable request to the corresponding author.
