# Supplementary material for: Dynamics of disease progression during treatment with Osimertinib in patients with EGFR T790M‐positive non‐small cell lung cancer
Source: Cancer Med. 2023 Apr 25;12(11):12285–98. doi: 10.1002/cam4.5926 (PMC10278531; doi:10.1002/cam4.5926)
Supplement: Supplementary file 1 — Table S1. [file CAM4-12-12285-s001.docx]

(Supplementary Table 1) Proportion of affected organs during osimertinib treatment.

| Metastatic organ at starting osimertinib | N | % | PD organ during osimertinib | N | % |
| --- | --- | --- | --- | --- | --- |
| Thoracic only  Brain  Brain meta only  Brain + 1 extracranial meta  Brain + 2 extracranial meta  Brain + 3 or more extracranial meta  Bone only  Liver only  1 other extracranial  2 other extracranial  3 or more other extracranial | 23  35  12  16  4  3  13  2  2  6  3 | 27.4  41.7  14.3  19.0  4.8  3.6  15.5  2.4  2.4  7.1  3.6 | Thoracic only  Brain  Brain meta only  Brain + 1 extracranial meta  Brain + 2 extracranial meta  Brain + 3 or more extracranial meta  Bone only  Liver only  1 other extracranial  2 other extracranial  3 or more other extracranial | 38  18  6  8  1  3  11  1  1  12  3 | 45.2  21.4  7.1  9.5  1.2  3.6  13.1  1.2  1.2  14.3  3.6 |

PD, progressive disease
