# Supplementary figures and images for: Dynamics of disease progression during treatment with Osimertinib in patients with EGFR T790M‐positive non‐small cell lung cancer
Source: Cancer Med. 2023 Apr 25;12(11):12285–98. doi: 10.1002/cam4.5926 (PMC10278531; doi:10.1002/cam4.5926)

## Slide 1
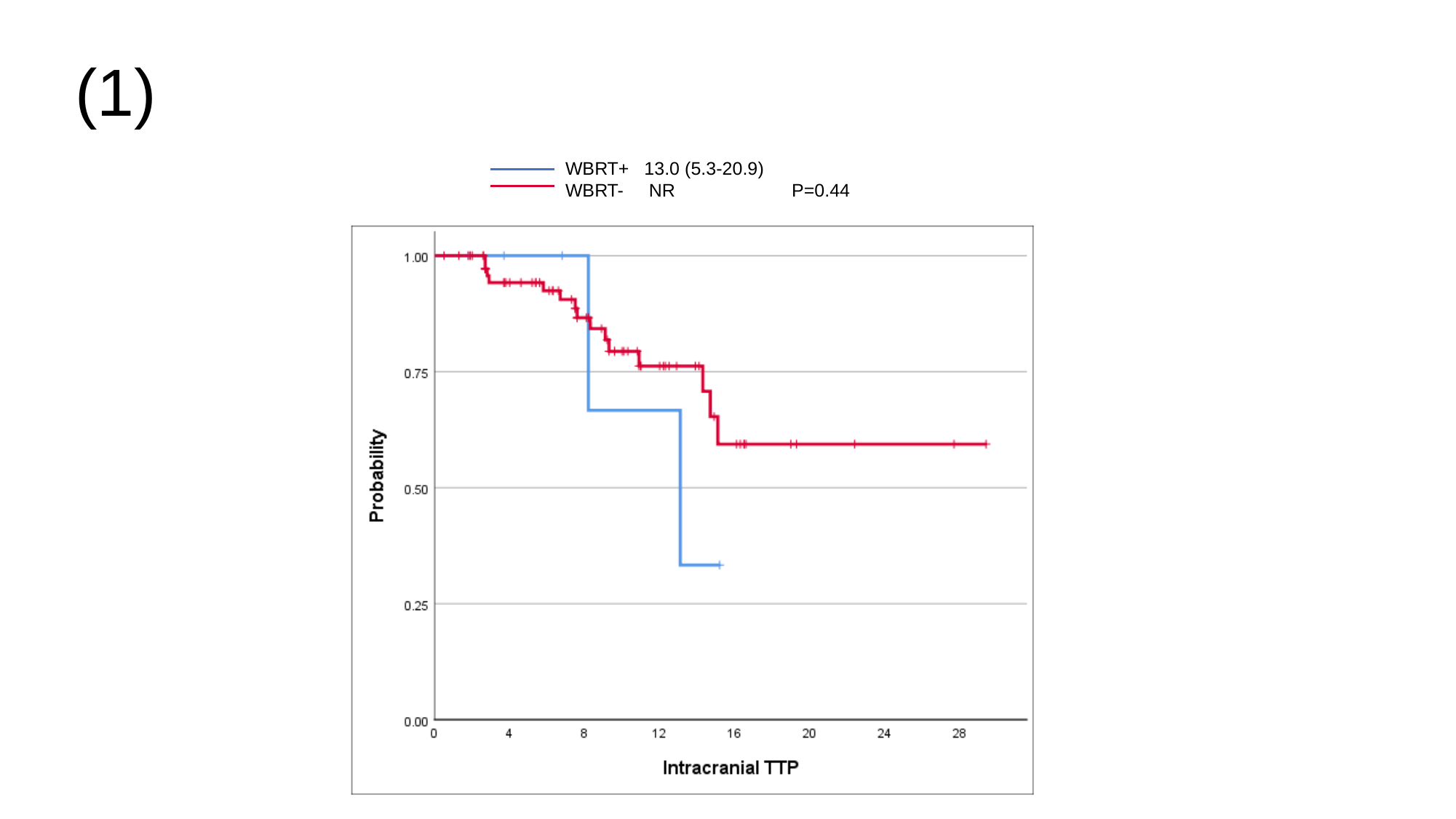

(1)
WBRT+ 13.0 (5.3-20.9)
WBRT- NR P=0.44

Supplement: Supplementary file 2 — Figure S1. [file CAM4-12-12285-s002.pptx]
